# A chimeolysin with extended-spectrum streptococcal host range found by an induced lysis-based rapid screening method

**DOI:** 10.1038/srep17257

**Published:** 2015-11-26

**Authors:** Hang Yang, Sara B. Linden, Jing Wang, Junping Yu, Daniel C. Nelson, Hongping Wei

**Affiliations:** 1 Key Laboratory of Special Pathogens and Biosafety, Center for Emerging Infectious Diseases, Wuhan Institute of Virology, Chinese Academy of Sciences, Wuhan 430071, China; 2 Institute for Bioscience and Biotechnology Research, University of Maryland, Rockville, MD 20850, USA; 3 Department of Veterinary Medicine, University of Maryland, College Park, MD 20742, USA

## Abstract

The increasing emergence of multi-drug resistant streptococci poses a serious threat to public health worldwide. Bacteriophage lysins are promising alternatives to antibiotics; however, their narrow lytic spectrum restricted to closely related species is a central shortcoming to their translational development. Here, we describe an efficient method for rapid screening of engineered chimeric lysins and report a unique “chimeolysin”, ClyR, with robust activity and an extended-spectrum streptococcal host range against most streptococcal species, including *S. pyogenes*, *S. agalactiae*, *S. dysgalactiae*, *S. equi*, *S. mutans*, *S. pneumoniae*, *S. suis* and *S. uberis*, as well as representative enterococcal and staphylococcal species (including MRSA and VISA). ClyR is the first lysin that demonstrates activity against the dominant dental caries-causing pathogen as well as the first lysin that kills all four of the bovine mastitis-causing pathogens. This study demonstrates the success of the screening method resulting in a powerful lysin with potential for treating most streptococcal associated infections.

*Streptococcus* spp. cause diseases ranging from mild sore throat to severe systemic infections^1^,^2^. Streptococcal pathogens with the highest disease burden in medical settings include beta-hemolytic *S. pyogenes* (i.e., group A streptococci, GAS) and *S. agalactiae* (i.e. group B streptococci, GBS), as well as alpha-hemolytic *S. pneumoniae* and the viridans streptococci (i.e. *S. mutans*, *S. mitis* and *S. sanguis*), while streptococci that infect livestock include *S. dysgalactiae* (i.e. group C streptococci, GCS), *S. agalactiae*, *S equi* (GCS), *S. suis*, and *S. uberis*. The increasing emergence of antibiotic-resistant streptococci poses a serious threat to public health worldwide^3^,^4^, which makes developing novel antibiotic alternatives imperative.

Lysins are encoded in the genomes of bacteriophage and function to digest the bacterial host cell wall for the release of progeny phage. This process kills the host bacteria, which can be replicated by addition of lysins to sensitive Gram-positive bacteria in the absence of phage, thereby representing a promising alternative to antibiotics^5^. The efficacy of lysins to clear several of these streptococcal species from mucous membrane surfaces or systemic infections in mice is well documented. For instance, the PlyC lysin has been used successfully to treat *S. pyogenes* infections^6^, PAL and Cpl-1 have been shown to prevent or treat *S. pneumoniae* lung infections^7^,^8^,^9^, and PlyGBS effectively protected mice against vaginal colonization by *S. agalactiae*^10^.

However, the narrow spectrum of lytic activity displayed by many of these enzymes presents a major limitation to pharmaceutical development given the realities and costs associated with obtaining regulatory approval for each new protein therapeutic^11^,^12^. For example, the host range of PAL and Cpl-1 is almost exclusive to *S. pneumoniae* (Supplementary Table S1). In addition, lysins displaying significant activity toward *S. mutans*, the etiological agent of dental caries, have not been reported. Thus, identification of lysins with high activity against an expanded host range, thereby increasing potential therapeutic applications, is needed to overcome financial hurdles associated with pharmaceutical development of this class of enzyme.

Lysins encoded by phages that infect Gram-positive hosts usually contain two distinct domains, typically an N-terminal catalytic domain (CD) and a C-terminal cell-wall binding domain (CBD)^13^. Because of the modular structure of lysins, chimeric lysins, or “chimeolysins”, can be constructed by shuffling these two domains from various natural lysins to obtain improved lytic activity^14^, enhanced solubility^15^, or an extended lytic spectrum^16^. Therefore, engineering chimeolysins represents an opportunity for finding novel enzymes with improved properties^17^. However, the current methods for developing chimeolysins depend largely on a trial-and-error approach, such as domain shuffling^15^,^16^,^18^ or an expressional-based strategy^19^, which limits the efficiency for discovering ideal chimeolysins. Furthermore, the properties of a chimeolysin (e.g., activity, solubility) are unpredictable and can only be established after recombinant protein engineering, expression, and purification.

In the present study, we report a new rapid screening method for generation of chimeolysins. Our approach combines expression of a chimeolysin library, constructed from donor CD and CBD domains, in an *E. coli* expression system combined with the aid of a novel enzyme, ClyN, which causes temporally controlled lysis of *E. coli* cells. Release of active chimeolysins by ClyN allows for screening of lytic activity against target species directly on culture plates (Supplementary Fig. S1). Using this method, we discovered and subsequently characterized a unique chimeolysin, ClyR. We demonstrate that ClyR has broad-spectrum lytic activity against most streptococcal species and is protective in an *in vivo* bacteremia model.

## Results

### Development of an induced releasing system in *E. coli*

**Figure 1:**
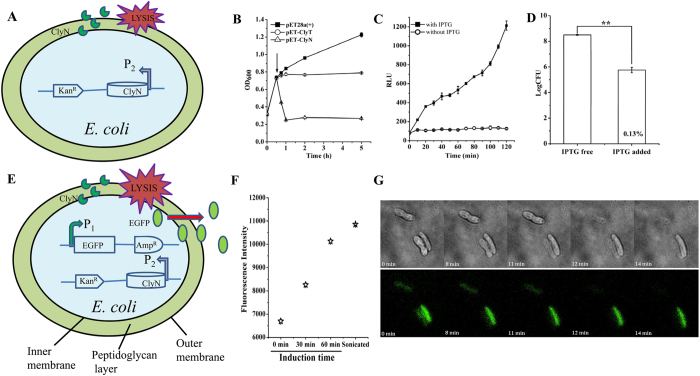
Induced lysis of host *E. coli* cells. (**A**) Schematic of the induced lysis of *E. coli* cells. (**B**) Effect of lytic proteins on the growth of host *E. coli* cells. BL21(DE3)/pET28a(+), BL21(DE3)/pET-*clyT*, and BL21(DE3)/pET-*clyN* cells were cultured in LB to OD_600_ = 0.8, then induced with 0.5 mM IPTG (arrow shown in the graph) and the changes of OD_600_ were monitored. (**C**) Release of ATP efflux from BL21(DE3)/pET-*clyN* cells after induction determined by a luciferin-luciferase system measuring relative light units (RLUs). Cells without IPTG induction were used as controls. (**D**) The lytic efficacy of ClyN induced lysis on host *E. coli* cells was confirmed by plating onto LB agar plates. (**E**) Schematic for the induced releasing of a recombinant protein (i.e. EGFP) inside *E. coli* cells. (**F**) Time-release kinetics of EGFP from ClyN induced lysis in host *E. coli* cells compared to sonicated cells. (**G**) Time-lapse microscopy analysis of ClyN induced host cell lysis. BL21(DE3)/pET-*clyN*/pBAD-*egfp* cells were induced with 0.2% L-arabinose, transferred to agar plates with 1 mM IPTG, then observed on a DeltaVision OMX V4 imaging system.

Initial efforts to randomly clone and express chimeolysins identified two fusions, ClyT and ClyN, which were notable for observed lethality to *E. coli* host cells upon induction (Fig. 1A). Expression of ClyT retarded growth of host cell resulting in a stable turbidity, while the induced expression of ClyN caused a rapid decrease in turbidity, with complete lysis occurring within 45 min (Fig. 1B). An ATP efflux was detected by a luciferin-luciferase system after induction of ClyN (Fig. 1C), indicating that lysis of the host cell from within led to the rapid decrease in turbidity concomitant with release of intracellular ATP. We also tested the influence of inductor (IPTG) concentration on the rate of lysis and found that 0.5 mM IPTG was strong enough to induce the lysis of host cell within 30 min (Supplementary Fig. S2). Quantifying bacterial counts before and after IPTG induction showed that the lytic efficacy of ClyN was as high as 99.87% (Fig. 1D). These findings allowed us to construct a system whereby a gene of interest could be expressed cytoplasmically by one promoter and release to the extracellular environment could be controlled by induction of ClyN on a separate promoter. To test this system as well as measure the releasing efficacy of recombinant proteins from lysed *E. coli*, we used EGFP as an indicator (Fig. 1E). An obvious increase of fluorescence in supernatants was noted after induction with IPTG in a time-dependent manner (Fig. 1F). Total fluorescence intensity in the supernatant after induction for 60 min was 89.1% of control cells disrupted using ultrasonication (Fig. 1F).

Finally, ClyN-induced lysis of host *E. coli* cells was further visualized by time-lapse microscopy. After expression of EGFP was induced, the BL21(DE3)/pET-*clyN*/pBAD-*egfp* cells were put onto LB-agar pads in the presence of 1 mM IPTG. At first, obvious green fluorescence inside individual cells could be seen in the microscopic field due to the existence of EGFP (Fig. 1G). Lysis of the host *E. coli* cells could be observed clearly within minutes, coupled with the dissipation of fluorescence and the disintegration of the whole cell structure (Fig. 1G and Supplementary Movie S1). A detailed view showed that the lysis was mostly completed through a process of deformation in the cell wall resulting in ultimate cell lysis (Supplementary Movie S2). This observation was quite consistent with the procedure of lysis from within caused by holin/lysin systems^20^.

### Rapid screening of chimeolysins

**Figure 2:**
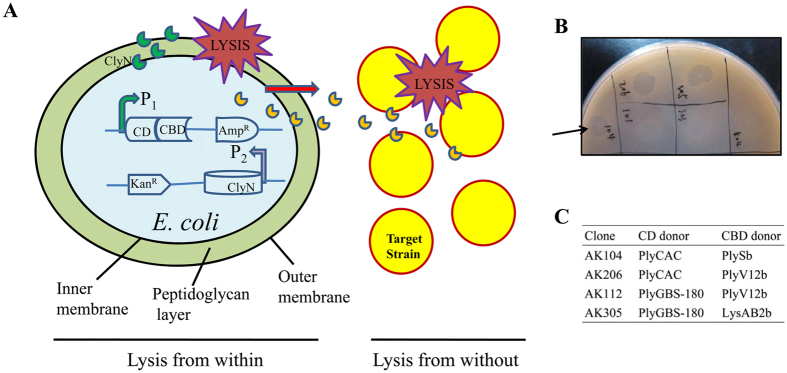
Screening of bactericidal chimeolysins. (**A**) Schematic of the screening method. (**B**) Clones were cultured with 0.2% L-arabinose overnight and screened for their capacity to generate clearing zones on soft agar plates overlaid with *S. dysgalactiae*, as indicated by arrow. (**C**) Identity of CD and CBD donors for clones identified as lytic when screened against *S. dysgalactiae*.

The rapid screening system was based on the combination of two classical types of lysis, known as lysis from within (i.e. ClyN-mediated lysis) and lysis from without (i.e. chimeolysin-mediated lysis) (Fig. 2A). A chimeolysin library containing combinations of seven CD donors and three CBD donors was built with the pBAD24 plasmid, cotransformed with the pET-*clyN* plasmid, and applied to plates for subsequent screening against target bacterial species (Supplementary Fig. S1). A great diversity in size and completeness of clearing zone was observed among these clones in plates when overlaid with *S. dysgalactiae* (Supplementary Fig. S3). Most of the clones had a minor inhibitory effect on the growth of *S. dysgalactiae* cells, displaying small, opaque zones on agar plates. In contrast, clone AK104 formed a large, clear zone in the *S. dysgalactiae* lawn (Fig. 2B). All clones showing lytic activity on *S. dysgalactiae* agar plates were sequenced to determine the combinations of the CDs and CBDs of these active chimeolysins (Fig. 2C).

### Enzymatic characteristics of ClyR

**Figure 3:**
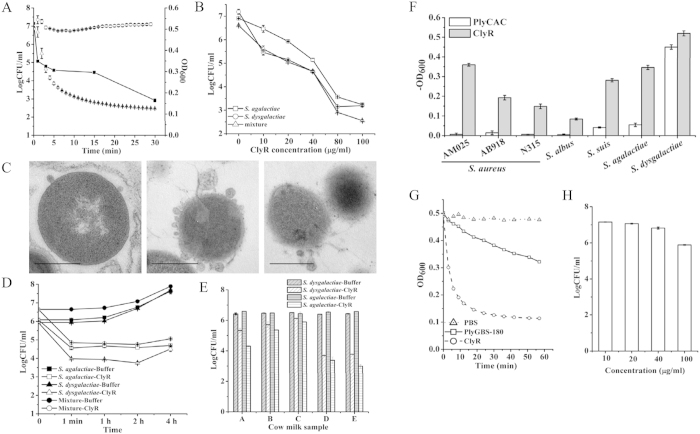
High lytic activity of ClyR. (**A**) Time-kill curves of ClyR against *S. dysgalactiae* ATCC 35666. Bacterial cells were washed once with PBS, treated with 25 μg/ml ClyR, and the change of OD_600_ (right Y-axis) were monitored by a microplate reader at 37 °C for 30 min. Triangles: ClyR; Circles: PBS controls. In parallel, the viable cell numbers (squares, left Y-axis) were calculated by plating onto BHI agar plates at different time points. (**B**) Dose-dependent lytic efficacy of ClyR against *S. agalactiae* S12, *S. dysgalactiae* ATCC 35666, or an equal mixture of both strains in market pasteurized milk at 30 °C for 1 h. (**C**) TEM images of *S. dysgalactiae* ATCC 35666 cells exposed to ClyR. Bar sizes: 500 nm. (**D**) Time-killing efficacy of ClyR (40 μg/ml) against *S. agalactiae* S12, *S. dysgalactiae* ATCC 35666, or an equal mixture of both strains in market pasteurized milk at 30 °C. (**E**) Lytic efficacy of ClyR (40 μg/ml) in pasteurized cow’s milk against *S. agalactiae* S12, or *S. dysgalactiae* ATCC 35666 at 30 °C for 1 h. Fresh cow milk samples A, B, and C were taken from healthy cows, and samples D and E were from mastitic cows. (**F**) Comparison of the activity of 0.89 μM ClyR or PlyCAC against various strains. (**G**) Comparison of the activity of 0.89 μM ClyR or PlyGBS-180 against *S. dysgalactiae* ATCC 35666. (**H**) Efficacy of PlyGBS-180 against *S. dysgalactiae* ATCC 35666 in market pasteurized milk at 30 °C for 60 min.

Clone AK104, containing a chimeolysin we named ClyR, showed the highest lytic activity against *S. dysgalactiae* on screening plates (Fig. 2B), and was therefore selected for further characterization. ClyR is well expressed as a soluble protein in *E. coli* and purification of >95% could be achieved as observed by 12% SDS-PAGE gel (Supplementary Fig. S4A). As shown in Fig. 3A, ClyR rapidly kills *S. dysgalactiae*; the turbidity decreased from 1.0 to near 0.2 within 30 min when treated with 25 μg/ml ClyR, corresponding to a 4 log reduction in CFU. Notably, a reduction of over 2 logCFU was observed in the first minute.

The influence of EDTA, temperature, and pH on the enzymatic activity of ClyR was also evaluated. Our results indicate that high concentrations of EDTA (up to 50 mM) only mildly affected ClyR activity (Supplementary Fig. S4B), differing from other reported lysins, many of which are calcium-dependent and susceptible to low concentrations of EDTA^21^. The suitable pH range for ClyR was from pH 5 to 11, with a maximal activity at pH 8 (Supplementary Fig. S4C). High lytic activity of ClyR was observed at temperatures below 40 °C (Supplementary Fig. S4D), which is consistent with many other lysins derived from phage that infect mesophilic organisms^22^,^23^,^24^. Meanwhile, high concentrations of NaCl had only a minor effect on the lytic activity of ClyR, with 84.5% relative activity retained in 1 M NaCl (Supplementary Fig. S4E). We also tested the stability of ClyR and found that this enzyme retained 78.7% and 93.6% lytic activity after storing at 4 °C for 2 months and −20 °C for 6 months, respectively (Supplementary Fig. S4F).

TEM analysis revealed that *S. dysgalactiae* cells exposed to ClyR for seconds suffered loss of peptidoglycan integrity allowing membrane extrusion and rapid disruption at single or multiple sites, resulting in the partial or total loss of cytoplasmic contents (Fig. 3C), validating the ClyR’s rapid lytic activity noted above. This observation is consistent with the typical phenomenon of osmosis-mediated cell lysis following the actions of phage lysins on target peptidoglycan reported elsewhere^15^.

### Efficacy of ClyR in milk

Because ClyR displays high lytic activity against *S. dysgalactiae*, we evaluated the bacteriolytic properties of ClyR against this organism and other mastitis-causing streptococci in milk. As shown in Fig. 3B, a dose-dependent response in market pasteurized milk was observed against *S. dysgalactiae*, *S. agalactiae*, and in a mixed culture. A reduction of ~1 logCFU was observed when treated with a low concentration of ClyR (10 μg/ml), while 80 μg/ml ClyR caused a reduction of over 5 logCFU. Next, we examined the time-killing efficacy of ClyR (40 μg/ml) against streptococci in market pasteurized milk. Results showed that the streptolytic process was almost complete in 1 minute, resulting in a reduction of over 2 logCFU (Fig. 3D). The rapid lytic efficacy of ClyR in milk was consistent with its performance in buffer (Fig. 3A). We further tested the efficacy of ClyR in pasteurized cow milk samples from healthy and mastitic cows. As shown in Fig. 3E, a 1–3 logCFU reduction was observed in ClyR-treated *S. dysgalactiae* and *S. agalactiae* cultures. Interestingly, the efficacy of ClyR-killing against streptococci in mastitic milk (samples D and E) was much higher than that in healthy milk (samples A-C). These data collectively show that ClyR works well in milk, with a rapid streptolytic efficacy similar to that of buffer conditions. ClyR represents a powerful lysin that could be active in comprehensive environments, and thus has the potential to be used as a therapeutic agent in anti-mastitis applications.

### Comparison the lytic activity of ClyR with other lysins

To further evaluate ClyR, we compared its lytic activity with two other well-known streptococcal lysin CDs, PlyCAC and PlyGBS-180. PlyCAC is the CD donor of ClyR, which has been crystallized^25^ and displays activity against select streptococci (Supplementary Table S1). The lysin PlyGBS, which is identical to the B30 lysin in the N-terminal 180 amino acids, was reported to have high lytic activity against *S. agalactiae*^10^. Truncation analysis revealed that the N-terminal 182 amino acids of B30 had much higher lytic activity than the full length enzyme against *S. dysgalactiae* and *S. agalactiae*^26^. We therefore cloned the N-terminal domain (amino acids 1–180) of lysin PlyGBS (PlyGBS-180) and compared its activity to ClyR. PlyCAC and PlyGBS-180 are well expressed in *E. coli* as shown in Supplementary Fig. S4A. To compare the lytic activity of ClyR and PlyCAC, we tested four species of staphylococci and three species of streptococci, and found that the lethal lytic activity of ClyR toward *S. dysgalactiae* was similar to that of PlyCAC. However, the lytic activity of ClyR against staphylococci, including two methicillin-resistant *S. aureus* (MRSA) strains, *S. suis*, and *S. agalactiae* was much higher than that of PlyCAC (Fig. 3F). The lytic activity of PlyGBS-180 against *S. dysgalactiae* was much lower than that of ClyR in buffer (Fig. 3G), which is further apparent when comparing dose-response curves between these two lysins (Supplementary Fig. S5). Moreover, the dose-dependent efficacy of PlyGBS-180 killing toward *S. dysgalactiae* in milk was markedly decreased compared to ClyR (Fig. 3H). At 40 μg/ml, PlyGBS-180 did not display any activity and only at 100 μg/ml there was a minor decrease of less than 1 logCFU observed.

### Lytic spectrum of ClyR

**Figure 4:**
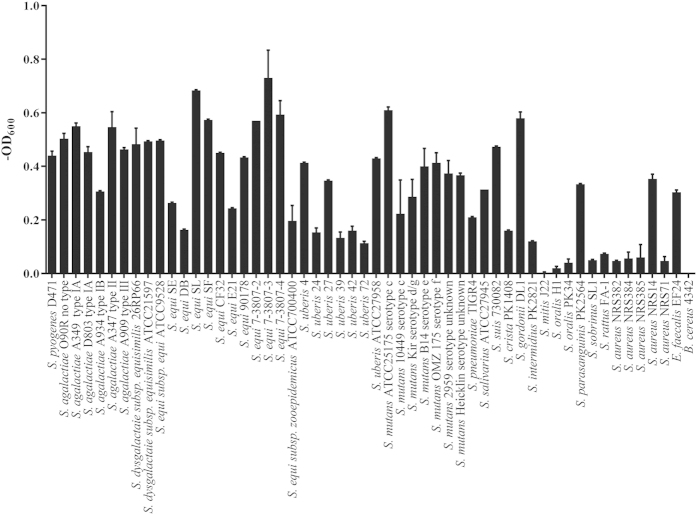
Lytic profile of ClyR. Multiple strains of streptococci (including *S. pyogenes*, *S. agalactiae*, *S. dysgalactiae*, *S. equi*, *S. uberis*, *S. mutans*, *S. pneumoniae*, *S. suis*, and *S. oralis*), *S. aureus*, *E. faecalis* and *B. cereus* were tested for susceptibility to ClyR. Strains were washed once with PBS and resuspended to a final OD_600_ of 0.8–1.2. After treating with 25 μg/ml of ClyR at 37 °C for 20 min, the final in OD_600_ were subtracted from the PBS treated control well to yield the net change in OD_600_. Experiments were done in triplicate and the error bars show the standard error.

In order to ascertain the lytic profile of ClyR, we tested the susceptibilities of various pathogenic bacteria to ClyR, including 19 species of streptococci and one species each of staphylococci, enterococci, and bacilli (Fig. 4). The turbidity reduction analysis revealed that ClyR was highly active against all streptococcal strains tested, including *S. pyogenes*, *S. agalactiae*, *S. dysgalactiae*, *S. equi*, *S. uberis*, *S. mutans*, *S. pneumoniae*, *S. suis*, *S. crista*, *S. gordonii*, *S. intermidius*, and *S. parasanguinis*. ClyR is effectively bactericidal to enterococci and has less, but still measurable, activity against staphylococci. Appreciably, ClyR’s lytic activity toward NRS14 (a vancomycin-intermediate resistant strain, VISA) is higher than non-VISA strains. These results demonstrate that ClyR has broad activity against streptococci and related species.

### Protective efficacy in a mouse infection model

**Figure 5:**
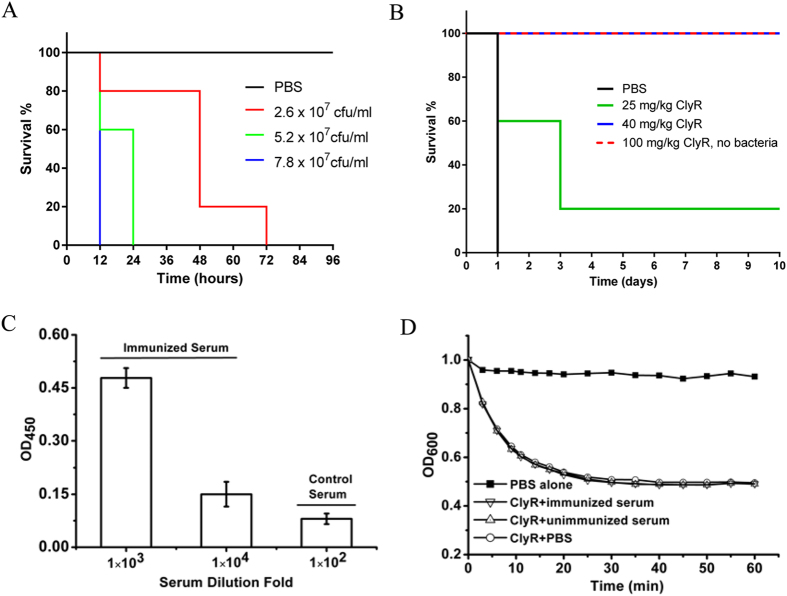
Protection efficacy of ClyR in an *S. agalactiae* mouse infection model. (**A**) Mice were intraperitoneally injected with different concentrations of S. *agalactiae* S12 cells and the survival rate of each group (5 each) was observed for three days. (**B**) Protection efficacy of ClyR in mice infected with 5.2 × 10^7^ CFU of S. *agalactiae* S12. Three hours post-injection, animals were divided into three groups randomly. Two groups (5 animals each) received 25 and 40 mg/kg ClyR intraperitoneally, respectively, and a third group (n = 5) was injected with PBS buffer. A fourth group (n = 5) that did not receive *S. agalactiae* injection received 100 mg/kg ClyR only as a safety/toxicity control. (**C**) ELISA test of ClyR-immunized serum. (**D**) Effect of ClyR-immunized serum on the lytic activity of ClyR against S. *agalactiae* S12 *in vitro*. The nonimmunized mouse serum was used as negative control.

The *in vivo* efficacy of ClyR was examined in a systemic *S. agalactiae* infection model. First, the lethality of a clinical isolate, *S. agalactiae* strain S12, was determined to elucidate inoculation dose for the model. Results showed that a single injection of 7.8 × 10^7^ CFU/mouse caused 100% death within 12 h, and a single injection of 5.2 × 10^7^ CFU/mouse caused 100% death within 24 h (Fig. 5A). In order to determine the protective efficacy of ClyR, we chose a dose of 5.2 × 10^7^ CFU/mouse for use in our systemic infection model. As shown in Fig. 5B, a dose-dependent protection was observed in the ClyR treated group, with 25% of the mice surviving 10 days when treated with 25 mg/kg ClyR and 100% surviving when the dose was increased to 40 mg/kg. Further tests showed that a single administration of a high dose (100 mg/kg) (Fig. 5B), or a continuous low dose (25 mg/kg per day for five continuous days, data not shown) of ClyR alone had no effect on the survival rate and produced no adverse effects to the mice in terms of body weight or activity. Repeated injection of ClyR did produce an immune response in mice (Fig. 5C); however, the immunized serum was unable to neutralize the lytic activity of ClyR *in vitro* (Fig. 5D), indicating that either the dominant antibody epitope on ClyR does not interfere with its catalytic or binding functions, or that the affinity of ClyR for the bacterial surface is higher than that of the antibodies for ClyR. Taken together, the robust efficacy against multiple species of streptococci both *in vitro* and *in vivo* indicates that ClyR is a potential alternative treatment of streptococcal-associated infections.

## Discussion

Lysis of the host cells from within by bacteriophage is controlled by the holin-lysin system^27^. Holins are small membrane proteins that accumulate in the membrane until, at a genetically determined time, they form pores in the inner membrane of the infected cell^28^. Lysins, expressed and accumulated in the cytosol of host cell^20^, gain access to their peptidoglycan substrate via these pores and cause rapid cell lysis^29^. In the present study, a chimeolysin (ClyN) was constructed by fusing a LysM domain to the C-terminal of a lysozyme gene from *E. coli*, which causes the rapid lysis of the host *E. coli* cells from within independent of a holin protein (Fig. 1). A previous study showed that the *Bacillus amyloliquefaciens* phage endolysin^30^, which also contains a C-terminal LysM domain, could lyse *Pseudomonas aeruginosa* from without directly, indicating that LysM domain may interfere or disorganize the bacterial membrane. Time-lapse microscopy reveals that host *E. coli* cells rapidly lyse after expression of ClyN, allowing for immediate release of intracellular EGFP, corresponding to the disappearance of fluorescence (Fig. 1G and Supplementary Movies S1 and S2). From these results, we speculate that ClyN uses its LysM domain to potentially span the inner membrane allowing the catalytic domain accesses to its peptidoglycan substrate, thus giving rise to the observed lysis from within of the host *E. coli* cells. However, further studies are needed to detail the molecular mechanisms underlying the interaction and kinetics of ClyN and/or its LysM domain with cell membranes.

The screening method reported here makes it possible to rapidly screen active chimeolysins on-plate (Fig. 2A, Supplementary Fig. S1). With this method, clones harboring active lysins could be identified from thousands of clones by the presence of clearing zones on screening plates (Fig. 2B). In addition to chimeolysins active against Gram-positive bacteria described here, the system could be adapted to screen chimeolysins against Gram-negative bacteria. Notably, several lysins have been reported to cause lysis from without against Gram-negative pathogens as native proteins^31^, or modified by attachment of a trans-membrane peptide^19^.

Nearly all of the phage-encoded lysins that have been characterized to date possess a narrow lytic spectrum, often restricted to the species-specific host range of the parental phage, as is the case with the *Bacillus anthracis*-specific PlyG lysin^32^. Otherwise, the host range is limited to a few closely related species, such as the PlyGRCS lysin, which is only lytic on *S. aureus* and *S. epidermidis*^22^. While a narrow spectrum of lytic activity may be evolutionarily conserved in phage/lysin systems, it is a major limitation to pharmaceutical development of this class of enzyme given the realities and costs associated with obtaining regulatory approval for each new protein therapeutic^11^,^12^.

Although several chimeolysins active against streptococci have been reported previously^33^,^34^, certain enzymatic characteristics of ClyR distinguish it from others. Among these is the rapid and robust lytic activity of ClyR against streptococci both in buffer and milk. A 2 logCFU reduction of *S. dysgalactiae* could be observed upon treatment with ClyR for 1 min in PBS buffer (Fig. 3A) and in milk (Fig. 3B). Such a rapid activity has been described for several lysins in buffer conditions, such as the streptococcal lysins PlyC^6^ and PAL^35^, and the *Bacillus anthracis* lysin PlyG^32^, but not observed for any lysin in milk conditions, which is important for mastitis control applications. In contrast to ClyR, most lysins are inhibited or lose significant activity when tested in milk^26^,^36^. Further studies showed that the performance of ClyR was not affected by high concentrations of EDTA (Supplementary Fig. S3B) or NaCl (Supplementary Fig. S3E), differing from most other lysins which are usually ion-dependent (on divalent metals, such as calcium) and are inhibited by addition of EDTA or high concentrations of NaCl^21^,^37^. Moreover, ClyR could function well across a large pH range, from pH 5 to 11, while most lysins are usually active only in a pH range from 5 to 8, with one exception being the PlyPH lysin^38^. The robust lytic activity of ClyR was evident not only against multiple streptococcal species *in vitro*, but was also verified in a mouse model of bacteremia (Fig. 5). Therefore, these special characteristics of ClyR make it a potential streptolytic therapeutic in environments that render other lysins inactive.

Another outstanding characteristic of ClyR is its broad lytic spectrum. As shown in Fig. 4, ClyR was active against multiple species of streptococci (including *S. pyogenes*, *S. agalactiae*, *S. dysgalactiae*, oral streptococci, mastitis-causing *S. uberis*, and *S. pneumoniae*), enterococci, and staphylococci (including MRSA and VISA). Significantly, each species is associated with a different set of virulence determinants that manifest distinct infection niches. *S. pyogenes* causes prevalent infections of the skin and mucous membranes, including impetigo and streptococcal pharyngitis, as well as life-threatening conditions such as necrotizing fasciitis^39^,^40^. *S. pneumoniae* is responsible for many infections worldwide, from superficial acute otitis media to serious invasive diseases, such as pneumonia and meningitis^41^,^42^,^43^. *S. agalactiae* is an etiologic agent of neonatal disease in humans^44^, as well as a leading source of mastitis in milking cows along with *S. dysgalactiae*, *S. uberis*, and *S. aureus*. Finally, *S. mutans* and *S. sanguis* are the leading causes of dental caries^45^,^46^.

Our previous study showed that the CBD donor of ClyR (the CBD of PlySs2, PlySb) has a broad binding capacity against streptococci^47^. The extensive binding capability of PlySb may be one reason for the broad lytic spectrum of PlySs2^48^ and thus the broad host range of ClyR. Nonetheless, the lytic spectrum of ClyR toward streptococci is even more robust than PlySs2, with ClyR displaying significant activity against *S. mutans*, *S. pneumonia*, and enterococci, whereas PlySs2 has limited or negligible activity against these species (Supplementary Table S1). Because PlyCAC shows a narrow spectrum of activity against some related streptococci, but displays no lytic activity against staphylococci and other strains (Fig. 3F), the broad lytic spectrum of ClyR may be due to the unique combination of CD and CBD donors rather than a single domain. As shown in Supplementary Table S1, many streptococcal lysins possess activity on a few closely related streptococcal species, while it is noteworthy that ClyR is the most broadly active streptococcal lysin described.

To our knowledge, there has not been a report of any lysin that has appreciable activity against *S. mutans*. In the present study we tested numerous serotypes of *S. mutans* and found that ClyR had noteworthy activity against all of them (Fig. 4). *S. mutans*, which usually grows as a biofilm, is a significant periodontal pathogen responsible for dental caries, a condition that lacks effective therapeutic options^49^. Significantly, many lysins active against planktonic streptococci and staphylococci have also been reported to be highly active against their biofilms, such as the ClyR CD donor, PlyC^50^, and the ClyR CBD donor, PlySs2^51^. Thus, we have reason to speculate that ClyR may have a robust biofilm degradation efficacy against *S. mutans* biofilms and have potential to be used in anti-dental caries applications.

Another possible application of ClyR is treatment of mastitis in cows. The major pathogens involved in bovine mastitis are *S. uberis*, *S. aureus*, *S. agalactiae*, and *S. dysgalactiae*. Since no lysin has been shown to work against all four pathogens, most researchers believe that a cocktail approach would be needed. However, ClyR does possess activity against all four, eliminating the need for multiple lysins to be used (Fig. 4). Our study further confirms that ClyR is active in milk, inducing a greater than 2 logCFU reduction of either *S. agalactiae*, or *S. dysgalactiae* alone, or a mixture of both strains, within 1 min (Fig. 3D). In contrast, PlyGBS-180 only has a minor effect at very high concentrations (Fig. 3H). Furthermore, the efficacy of ClyR against streptococci was also examined in fresh cow milk samples. Interestingly, higher rates of killing were observed in mastitic cow milk samples than that of in the healthy milk samples (Fig. 3E), perhaps due to physiological differences in milk quality between the samples (i.e. pH, milk protein concentration, somatic cell count, etc.). In view of the measurable activity of ClyR against staphylococci, especially, the high lytic activity against NRS14 (a vancomycin-intermediate *S. aureus*) and AM025 (a methicillin-resistant *S. aureus*), ClyR exhibits potential capability in anti-mastitis applications.

ClyR additionally shows high lytic activity against both pneumococci and enterococci, a property not observed in other pneumococcal-targeted lysins. Only the lysins derived from pneumococcal phages have been shown to have significant activity on pneumococci. Moreover, the CBDs of these lysins either displays choline-binding repeats (i.e. PAL and Cpl-1)^52^, or contains three CW_7 repeats that recognize the bacterial cell wall in a choline-independent manner (i.e. Cpl-7)^53^,^54^. In contrast, the CBD of ClyR, which belongs to the SH3 superfamily, does not contain either of these repeats and shares a low identity to the CBDs of Cpl-1 (belongs to choline-binding glucan_65_rpt) and Cpl-7 (belongs to Cpl-7 superfamily). Therefore, the detailed molecular mechanism underlying the binding and lytic activity of ClyR against *S. pneumoniae* still needs further study.

In summary, we reported here a unique chimeolysin, ClyR, which has robust lytic activity and an extended-spectrum host range against multiple species of streptococci both *in vitro* and *in vivo*. Taking into account its strong stability, ClyR has great potential to be an effective therapeutic agent against streptococcal mediated infections. This study also provides, for the first time, an induced lysis-based rapid, visual screening method for finding active and soluble peptidoglycan hydrolases (i.e. lysins or chimeolysins) expressed by *E. coli*, which allows for on-plate screening offering an efficient way to develop bactericidal lysins against multi-drug resistant pathogens. The initial success of identifying the ClyR chimeolysin demonstrates the potential of the screening method.

## Methods

### Bacterial strains

Streptococcal strains were grown in Todd-Hewitt broth, supplemented with 1% yeast extract (THY) (Alpha Bioscience). *Escherichia coli* BL21(DE3), used for cloning and expression of recombinant proteins, was grown in Luria Broth (LB) medium. All other strains were grown in tryptic soy broth (TSB) (Alpha Bioscience). All bacterial strains (Supplementary Table S2) were grown at 37 °C. *S. agalactiae* strain S12 was isolated from a mastitic cow milk sample, identified by 16S rDNA sequencing analysis combined with biochemistry testing using a MicroStation system (Biolog, GEN III Omnilog Combo Plus System, USA).

### Construction of lytic proteins against *E. coli*

Two engineered proteins, ClyN and ClyT, were constructed and tested. ClyN was constructed by fusing the lysozyme gene from *E. coli* strain K-12 substrain MG1655 (GenBank No.: AIZ92304.1) with a C-terminal LysM domain cloned from lactococcal bacteriophage Tuc2009 lysin Lystuc2009 (GenBank No.: AF109874.2, 310–428 aa). The protein ClyT (from N-terminal to C-terminal) consists of the SSP0609 gene from *S. saprophyticus* subsp. *saprophyticus* ATCC 15305 (NCBI Reference Sequence: NC_007350.1) followed by the CBD of PlySs2 (147–245 aa) and the transmembrane fragments of *Acinetobacter baumannii* phage φAB2 lysin LysAB2 (113–185 aa). ClyN and ClyT encoding sequences were chemically synthesized by Gen Script Co., Ltd. (Nanjing, China) and cloned into the pET28a(+) plasmid. After verification by sequencing, the plasmid was transformed into *E. coli* BL21(DE3) for expression.

### Lytic efficacy of ClyN on *E. coli*

To test the effect of lytic proteins on the growth of host cell, recombinant *E. coli* cells BL21(DE3)/pET-*clyN* and BL21(DE3)/pET-*clyT* were cultured to an OD_600_ of ~0.8 and induced with various concentrations of isopropyl β-D-thiogalactoside (IPTG). The turbidity was monitored by a microplate reader (Synergy H1, BioTek, USA). ATP released from host BL21(DE3)/pET-*clyN* cells was determined by a luciferin-luciferase system. Briefly, cells at OD_600_ of ~0.8 were treated with 1 mM IPTG, 0.25 μM luciferase and 5 μM luciferin (containing 12.5 μM coenzyme A) in a 200 μl reaction volume. The relative light units (RLUs) were monitored by the microplate reader in 5-sec integrations. Samples without IPTG served as blank controls.

Lytic efficacy of ClyN induced lysis to host *E. coli* cells was further determined by a plating assay. BL21(DE3)/pET-*clyN* cells were cultured at 37 °C overnight. After dilution with IPTG in ddH_2_O (final IPTG concentration was 0.5 mM in each dilution), cells were plated on LB agar plates and grown at 37 °C overnight for enumeration of colonies. Dilutions without IPTG were used as controls.

### Induced releasing of recombinant EGFP

An enhanced green fluorescent protein (EGFP) coding fragment was cloned into pBAD24 plasmid (pBAD-*egfp*), and transformed into BL21(DE3) cells with pET-*clyN* plasmid. The resulting clone was inoculated in LB containing 0.2% L-arabinose overnight with 200 μg/ml ampicillin and 50 μg/ml kanamycin. Afterwards, cells were collected and induced with 0.5 mM IPTG for 0, 0.5, 1 and 2 h in fresh LB to induce cell lysis. Another group identically prepared and in the same volume were lysed directly by sonication (SCIENTZ IID: 37%, 30 min, 3 sec pulse/5 sec break steps) on ice. The fluorescence in the supernatant was detected by the microplate reader for each of treatments.

The induced lysis of host *E. coli* cells was further confirmed by time-lapse microscopy. BL21(DE3)/pET-*clyN*/pBAD-*egfp* cells were grown to mid-exponential phase in LB with 0.2% L-arabinose, washed twice with PBS and resuspended in fresh LB containing 1 mM IPTG. For imaging, cells were placed between LB-agar pads and a cover glass as described previously^55^. Images were acquired using a DeltaVision OMX V4 system (Applied Precision, GE Healthcare, USA) and resulting pictures were further processed with the WORX instrument software.

### Construction of chimeolysin library

A library was constructed consisting of three CBD and seven CD donors. Two typical CBDs from lysins with multi-genus lytic activity and a potential membrane permeable peptide were employed as CBD donors, CD donors contained seven CDs from various bacteriophage lysins, including two staphylococci phage lysins, two *Listeria* phage lysins and three streptococci phage lysins (Supplementary Table S3). All the genes of the CBD and CD donors were chemically synthesized by Gen Script Co., Ltd. (Nanjing, China) and amplified with primers (Supplementary Table S4) to introduce *Sma*I and *Xba*I sites for CD donors, and *Xba*I and *Sph*I sites for CBD donors. All the donor fragments were mixed with an equal mole ratio and cloned into *Sma*I and *Sph*I sites of pBAD24 randomly and transformed into *E. coli* BL21(DE3) cells. Subclones grown in the transformed plate were collected and prepared into competent recipient cells by a CaCl_2_ method following the instructions of a commercial kit (TaKaRa, Liaoning, China). Afterwards, purified pET-*clyN* plasmid was transformed into the competent cells obtained above and screened by plates with ampicillin (200 μg/ml) and kanamycin (50 μg/ml). The resulting double transformed library was stored at 4 °C for further selection of active chimeolysins.

### On-plate bactericidal chimeolysin screening

To select active chimeolysin against streptococci, individual subclones from the transformant library were inoculated into 1 ml fresh medium with 0.2% L-arabinose in wells of 24-well plates and grown overnight at 37 °C to allow the expression of chimeolysins. The next morning, IPTG was added into the wells at a final concentration of 0.5 mM to initiate expression of ClyN, and 5 μl of these cultures were spotted onto agar plates overlaid with *S. dysgalactiae* ATCC 35666 and incubated overnight at 37 °C. The subclones that caused large clearing zones in the agar plates were noted and the corresponding clones in the 24-well plates were picked for sequencing analysis to determine the identity of the cloned chimeolysin.

### Construction of plasmids

Sequence analysis revealed that strain AK104 expressed an engineered lysin consisting of the CHAP domain of the PlyC lysin (amino acids 314–465), called PlyCAC^25^, and the cell wall binding domain of the PlySs2 lysin (amino acids 147–245), called PlySb^48^. To further ascertain the enzymatic characteristics of this chimeolysin, which we named as ClyR, its coding sequence was cloned into pET28a(+) plasmid with primers ClyR-F and ClyR-R. In order to compare the lytic activity of ClyR with that of other related lysins, PlyCAC and PlyGBS-180 (the N-terminal 180 amino acids of lysin PlyGBS, GenBank: AAR99416.1) were also cloned into pET28a(+) plasmid. Primers used for PlyCAC cloning were ClyR-F and PlyCAC-R; for PlyGBS-180 cloning, primers were PlyGBS-180-F and PlyGBS-180-R. All final plasmids were transformed into *E. coli* BL21(DE3) after confirmation by sequencing.

### Protein purification

To purify lytic enzymes PlyCAC, PlyGBS-180 and ClyR, bacterial cells were induced with 0.5 mM IPTG overnight at 16 °C and collected for protein purification after sonication on ice. Protein was collected by washing and eluting with 60 and 250 mM imidazole through a nickel nitrilotriacetic acid column, respectively. Active fractions were pooled and dialyzed against PBS buffer (137 mM NaCl, 2.7 mM KCl, 4.3 mM Na_2_HPO_4_ · H_2_O, 1.4 mM KH_2_PO_4_, pH 7.4) and stored at −80 °C until needed.

### Lytic activity assay

The enzymatic activity was determined as described previously^6^, with minor modifications. Briefly, bacterial cells were harvested by centrifugation, resuspended in buffer (PBS, pH 7.4), and mixed in an equal volume with ClyR (25 μg/ml) to a final OD_600_ of 0.8–1.2. The optical density of the samples was recorded every 15 seconds for 20 minutes at 37 °C, and the final OD_600_ was noted. The susceptibility was presented as the difference of the decrease in OD_600_ between ClyR treated wells and PBS treated wells at the final time point and recorded as the drop in OD_600_ (i.e., –OD_600_).

The influence of EDTA, NaCl, pH and temperature on the activity of ClyR, was also evaluated. To accomplish this, *S. dysgalactiae* ATCC 35666 was suspended in phosphate buffer containing various concentrations of EDTA (ranging from 0 to 500 mM), or NaCl (ranging from 0 to 1000 mM), or suspended in a universal buffer with pH ranging from 2 to 12. The universal buffer was made by mixing equal parts of 20 mM boric acid and 20 mM phosphoric acid, followed by titration with sodium hydroxide as described^38^. After adding ClyR to a final concentration of 25 μg/ml, the decrease in OD_600_ was monitored in each well by a microplate reader (Synergy H1, BioTek, USA) at 37 °C for 60 min. To evaluate the influence of temperature, ClyR was stored at various temperatures ranging from 4 to 100 °C for 60 min and the residual enzymatic activity was tested in the same instrument under the same conditions. The lytic activity was measured as the decrease in OD_600_ within 30 min and normalized relative to the maximum activity in each condition. All experiments were performed in triplicate and bacterial cells treated with PBS were used as controls.

To compare the activity of ClyR with that of PlyCAC, mid-log-phase cultures of bacterial strains were pelleted and resuspended in PBS to a final OD_600_ of 1.0. Equal molar amount of each enzyme (0.89 μM) were mixed with a bacterial suspension (100 μl) and the decrease in OD_600_ was monitored by the microplate reader.

The action of ClyR on the cell wall was monitored by thin-section transmission electron microscopy (Tecnai G^2^ 20 TWIN, FEI,USA). *S. dysgalactiae* ATCC 35666 suspensions were incubated with 40 μg/ml of ClyR at 37 °C for 10, 30, or 60 seconds. The reactions were terminated by addition of 2.5% glutaraldehyde and the fixed samples were analyzed by TEM.

### Lytic activity in milk

To test the efficacy of ClyR in milk, *S. dysgalactiae* ATCC 35666 and *S. agalactiae* S12 were pre-mixed alone or together in market pasteurized milk or pasteurized fresh milk from mastitic and healthy milk cows. After treating with different concentrations of ClyR or PlyGBS-180 (0, 10, 20, 40, 80, or 100 μg/ml) at 30 °C for 1, 2, 3, or 4 h, the viable cell number was evaluated by plating 10 μl of the mixture onto Brain Heart Infusion (BHI) agar plates.

### Mouse protection experiment

All mouse experiments utilized an ABSL-2 lab and the methods were conducted in accordance with the approved regulations and guidelines set forth by the Animal Experiments Committee of Wuhan Institute of Virology, Chinese Academy of Sciences (approval number WIVA17201401). In the mouse systemic infection model, the minimal lethal dose (MLD) of *S. agalactiae* S12 was determined initially. Female BALB/c mice (6–8 weeks old) were randomly separated into 4 groups (5 each), three of which were injected intraperitoneally with different concentrations of *S. agalactiae* S12. The last group was challenged with PBS buffer as a control. For protective efficacy examination, mice were inoculated intraperitoneally with the dose of *S. agalactiae* S12 that caused 100% mortality within 24 h. Three hours after challenge, the mice were divided into 3 groups randomly. Two groups (5 each) received 25 or 40 mg/kg ClyR intraperitoneally, and the third group (n = 5) was injected with PBS buffer. To evaluate the toxicity of ClyR, another group of mice without infection received 100 mg/kg ClyR once (n = 5), or 25 mg/kg per day for five continuous days (n = 3). The survival rates for all groups were observed for 10 days.

### Immunological test

The neutralization effect of ClyR-specific antibodies on the activity of ClyR was tested using a standard immunological protocol as described^14^,^56^. Briefly, mice were immunized with ClyR (0.5 mg) and given two booster immunizations at ten days intervals to evoke specific antibodies. Mice sera were sampled 15 days after the last injection and the serum titers were checked by ELISA using horseradish peroxidase-conjugated goat anti-mouse IgG (QF-Bio, Shanghai, China). To test the neutralization effect, ClyR, at a final concentration of 12.5 μg/ml, was incubated with 80 μl of the ClyR immunized mouse serum (the titer was >10^4^) at 37 °C for 15 min. Afterwards, the lytic activity against *S. agalactiae* S12 was checked immediately, using non-immunized mouse serum and PBS as controls.

## Additional Information

**How to cite this article**: Yang, H. *et al.* A chimeolysin with extended-spectrum streptococcal host range found by an induced lysis-based rapid screening method. *Sci. Rep.* **5**, 17257; doi: 10.1038/srep17257 (2015).

## Supplementary Material

Supplementary Movie S1

Supplementary Movie S2

Supplementary Information

## References

[b1] Awada A., van der Auwera P., Meunier F., Daneau D. & Klastersky J. Streptococcal and enterococcal bacteremia in patients with cancer. Clin Infect Dis 15, 33–48 (1992). 1617072 10.1093/clinids/15.1.33

[b2] Tan L. K., Eccersley L. R. & Sriskandan S. Current views of haemolytic streptococcal pathogenesis. Curr Opin Infect Dis 27, 155–164, 10.1097/QCO.0000000000000047 (2014). 24573012

[b3] McCracken G. H. Jr. Emergence of resistant *Streptococcus pneumoniae*: a problem in pediatrics. Pediatr Infect Dis J 14, 424–428 (1995). 7638032 10.1097/00006454-199505001-00004

[b4] Kimura K. *et al.* High frequency of fluoroquinolone- and macrolide-resistant streptococci among clinically isolated group B streptococci with reduced penicillin susceptibility. J Antimicrob Chemother 68, 539–542, 10.1093/jac/dks423 (2013). 23111853

[b5] Rodriguez-Rubio L. *et al.* Phage lytic proteins: biotechnological applications beyond clinical antimicrobials. Crit Rev Biotechnol 1–11, 10.3109/07388551.2014.993587 (2015). 25603721

[b6] Nelson D., Loomis L. & Fischetti V. A. Prevention and elimination of upper respiratory colonization of mice by group A streptococci by using a bacteriophage lytic enzyme. Proc Natl Acad Sci USA 98, 4107–4112, 10.1073/pnas.061038398 (2001). 11259652 PMC31187

[b7] Jado I. *et al.* Phage lytic enzymes as therapy for antibiotic-resistant *Streptococcus pneumoniae* infection in a murine sepsis model. J Antimicrob Chemother 52, 967–973, 10.1093/jac/dkg485 (2003). 14613958

[b8] Entenza J. M., Loeffler J. M., Grandgirard D., Fischetti V. A. & Moreillon P. Therapeutic effects of bacteriophage Cpl-1 lysin against *Streptococcus pneumoniae* endocarditis in rats. Antimicrob Agents Chemother 49, 4789–4792, 10.1128/AAC.49.11.4789-4792.2005 (2005). 16251333 PMC1280127

[b9] McCullers J. A., Karlstrom A., Iverson A. R., Loeffler J. M. & Fischetti V. A. Novel strategy to prevent otitis media caused by colonizing *Streptococcus pneumoniae*. PLoS Pathog 3, e28, 10.1371/journal.ppat.0030028 (2007). 17381239 PMC1829406

[b10] Cheng Q., Nelson D., Zhu S. & Fischetti V. A. Removal of group B streptococci colonizing the vagina and oropharynx of mice with a bacteriophage lytic enzyme. Antimicrob Agents Chemother 49, 111–117, 10.1128/AAC.49.1.111-117.2005 (2005). 15616283 PMC538902

[b11] Conly J. & Johnston B. Where are all the new antibiotics? The new antibiotic paradox. Can J Infect Dis Med Microbiol 16, 159–160 (2005). 18159536 10.1155/2005/892058 PMC2095020

[b12] Cooper M. A. & Shlaes D. Fix the antibiotics pipeline. Nature 472, 32, 10.1038/472032a (2011). 21475175

[b13] Fischetti V. A. Bacteriophage lysins as effective antibacterials. Curr Opin Microbiol 11, 393–400, 10.1016/j.mib.2008.09.012 (2008). 18824123 PMC2597892

[b14] Yang H. *et al.* Novel chimeric lysin with high-level antimicrobial activity against methicillin-resistant *Staphylococcus aureus in vitro* and *in vivo*. Antimicrob Agents Chemother 58, 536–542, 10.1128/AAC.01793-13 (2014). 24189265 PMC3910767

[b15] Daniel A. *et al.* Synergism between a novel chimeric lysin and oxacillin protects against infection by methicillin-resistant *Staphylococcus aureus*. Antimicrob Agents Chemother 54, 1603–1612, 10.1128/AAC.01625-09 (2010). 20086153 PMC2849374

[b16] Dong Q. *et al.* Construction of a chimeric lysin Ply187N-V12C with extended lytic activity against staphylococci and streptococci. Microb Biotechnol 8, 210–220, 10.1111/1751-7915.12166 (2015). 25219798 PMC4353335

[b17] Yang H., Yu J. & Wei H. Engineered bacteriophage lysins as novel anti-infectives. Front Microbiol 5, 542, 10.3389/fmicb.2014.00542 (2014). 25360133 PMC4199284

[b18] Vipra A. A. *et al.* Antistaphylococcal activity of bacteriophage derived chimeric protein P128. BMC Microbiol 12, 41, 10.1186/1471-2180-12-41 (2012). 22439788 PMC3362776

[b19] Briers Y. *et al.* Engineered endolysin-based “Artilysins” to combat multidrug-resistant gram-negative pathogens. MBio 5, e01379–01314, 10.1128/mBio.01379-14 (2014). 24987094 PMC4161244

[b20] Young R. Bacteriophage lysis: mechanism and regulation. Microbiol Rev 56, 430–481 (1992). 1406491 10.1128/mr.56.3.430-481.1992 PMC372879

[b21] Becker S. C. *et al.* Lytic activity of the staphylolytic Twort phage endolysin CHAP domain is enhanced by the SH3b cell wall binding domain. FEMS Microbiol Lett 362, 1–8, 10.1093/femsle/fnu019 (2015). 25790497 PMC4811206

[b22] Linden S. B. *et al.* Biochemical and biophysical characterization of PlyGRCS, a bacteriophage endolysin active against methicillin-resistant *Staphylococcus aureus*. Appl Microbiol Biotechnol 99, 741–752, 10.1007/s00253-014-5930-1 (2015). 25038926

[b23] Filatova L. Y., Becker S. C., Donovan D. M., Gladilin A. K. & Klyachko N. L. LysK, the enzyme lysing *Staphylococcus aureus* cells: specific kinetic features and approaches towards stabilization. Biochimie 92, 507–513, 10.1016/j.biochi.2010.01.026 (2010). 20144680

[b24] Sanz J. M., Garcia J. L., Laynez J., Usobiaga P. & Menendez M. Thermal stability and cooperative domains of CPL1 lysozyme and its NH2- and COOH-terminal modules. Dependence on choline binding. J Biol Chem 268, 6125–6130 (1993). 8454587

[b25] McGowan S. *et al.* X-ray crystal structure of the streptococcal specific phage lysin PlyC. Proc Natl Acad Sci USA 109, 12752–12757, 10.1073/pnas.1208424109 (2012). 22807482 PMC3412044

[b26] Donovan D. M. *et al.* Peptidoglycan hydrolase fusions maintain their parental specificities. Appl Environ Microbiol 72, 2988–2996, 10.1128/AEM.72.4.2988-2996.2006 (2006). 16598006 PMC1448998

[b27] Young R. & Wang I. Phage lysis. Bacteriophage 2, 104–126 (2006).

[b28] Young R. & Blasi U. Holins: form and function in bacteriophage lysis. FEMS Microbiol Rev 17, 191–205, 0168-6445(94)00079-4 (1995). 7669346 10.1111/j.1574-6976.1995.tb00202.x

[b29] Wang I. N., Smith D. L. & Young R. Holins: the protein clocks of bacteriophage infections. Annu Rev Microbiol 54, 799–825, 10.1146/annurev.micro.54.1.799 (2000). 11018145

[b30] Morita M. *et al.* Functional analysis of antibacterial activity of *Bacillus amyloliquefaciens* phage endolysin against Gram-negative bacteria. FEBS Lett 500, 56–59 (2001). 11434926 10.1016/s0014-5793(01)02587-x

[b31] Lood R. *et al.* Novel phage lysins capable of killing the multidrug resistant Gram-negative bacterium *Acinetobacter baumannii* in a mouse sepsis model. Antimicrob Agents Chemother, 10.1128/AAC.04641-14 (2015). PMC4356752 25605353

[b32] Schuch R., Nelson D. & Fischetti V. A. A bacteriolytic agent that detects and kills *Bacillus anthracis*. Nature 418, 884–889, 10.1038/nature01026 (2002). 12192412

[b33] Diez-Martinez R. *et al.* A novel chimeric phage lysin with high *in vitro* and *in vivo* bactericidal activity against *Streptococcus pneumoniae*. J Antimicrob Chemother, 10.1093/jac/dkv038 (2015). 25733585

[b34] Diaz E., Lopez R. & Garcia J. L. Chimeric phage-bacterial enzymes: a clue to the modular evolution of genes. Proc Natl Acad Sci USA 87, 8125–8129 (1990). 1978320 10.1073/pnas.87.20.8125 PMC54905

[b35] Loeffler J. M., Nelson D. & Fischetti V. A. Rapid killing of *Streptococcus pneumoniae* with a bacteriophage cell wall hydrolase. Science 294, 2170–2172, 10.1126/science.1066869 (2001). 11739958

[b36] Celia L. K., Nelson D. & Kerr D. E. Characterization of a bacteriophage lysin (Ply700) from *Streptococcus uberis*. Vet Microbiol 130, 107–117, 10.1016/j.vetmic.2007.12.004 (2008). 18242012

[b37] Gu J. *et al.* Structural and biochemical characterization reveals LysGH15 as an unprecedented “EF-hand-like” calcium-binding phage lysin. PLoS Pathog 10, e1004109, 10.1371/journal.ppat.1004109 (2014). 24831957 PMC4022735

[b38] Yoong P., Schuch R., Nelson D. & Fischetti V. A. PlyPH, a bacteriolytic enzyme with a broad pH range of activity and lytic action against *Bacillus anthracis*. Journal of Bacteriology 188, 2711–2714, 10.1128/Jb.188.7.2711-2714.2006 (2006). 16547060 PMC1428399

[b39] Bisno A. L. Group A streptococcal infections and acute rheumatic fever. N Engl J Med 325, 783–793, 10.1056/NEJM199109123251106 (1991). 1870652

[b40] Esposito S. *et al.* Geoepidemiological hints about *Streptococcus pyogenes* strains in relationship with acute rheumatic fever. Autoimmun Rev, 10.1016/j.autrev.2015.03.001 (2015). 25772310

[b41] Grabenstein J. D. & Weber D. J. Pneumococcal serotype diversity among adults in various countries, influenced by pediatric pneumococcal vaccination uptake. Clin Infect Dis 58, 854–864, 10.1093/cid/cit800 (2014). 24344141

[b42] Corrales-Medina V. F., Musher D. M., Shachkina S. & Chirinos J. A. Acute pneumonia and the cardiovascular system. Lancet 381, 496–505, 10.1016/S0140-6736(12)61266-5 (2013). 23332146

[b43] Musher D. M., Rueda A. M., Kaka A. S. & Mapara S. M. The association between pneumococcal pneumonia and acute cardiac events. Clin Infect Dis 45, 158–165, 10.1086/518849 (2007). 17578773

[b44] Farley M. M. *et al.* A population-based assessment of invasive disease due to group B Streptococcus in nonpregnant adults. N Engl J Med 328, 1807–1811, 10.1056/NEJM199306243282503 (1993). 8502269

[b45] Simon-Soro A. & Mira A. Solving the etiology of dental caries. Trends Microbiol 23, 76–82, 10.1016/j.tim.2014.10.010 (2015). 25435135

[b46] Loesche W. J. Role of *Streptococcus mutans* in human dental decay. Microbiol Rev 50, 353–380 (1986). 3540569 10.1128/mr.50.4.353-380.1986 PMC373078

[b47] Huang Y., Yang H., Yu J. & Wei H. Molecular dissection of phage lysin PlySs2: integrity of the catalytic and cell wall binding domains is essential for its broad lytic activity. Virol Sin 30, 45–51, 10.1007/s12250-014-3535-6 (2015). 25680444 PMC8200883

[b48] Gilmer D. B., Schmitz J. E., Euler C. W. & Fischetti V. A. Novel bacteriophage lysin with broad lytic activity protects against mixed infection by *Streptococcus pyogenes* and methicillin-resistant *Staphylococcus aureus*. Antimicrob Agents Chemother 57, 2743–2750, 10.1128/AAC.02526-12 (2013). 23571534 PMC3716137

[b49] Kranz A. M. *et al.* Preventive services by medical and dental providers and treatment outcomes. J Dent Res 93, 633–638, 10.1177/0022034514536731 (2014). 24891593 PMC4107553

[b50] Shen Y., Koller T., Kreikemeyer B. & Nelson D. C. Rapid degradation of *Streptococcus pyogenes* biofilms by PlyC, a bacteriophage-encoded endolysin. J Antimicrob Chemother 68, 1818–1824, 10.1093/jac/dkt104 (2013). 23557924

[b51] Schuch R. *et al.* Combination therapy with lysin CF-301 and antibiotic is superior to antibiotic alone for treating methicillin-resistant *Staphylococcus aureus*-induced murine bacteremia. J Infect Dis 209, 1469–1478, 10.1093/infdis/jit637 (2014). 24286983 PMC3982849

[b52] Hermoso J. A. *et al.* Structural basis for selective recognition of pneumococcal cell wall by modular endolysin from phage Cp-1. Structure 11, 1239–1249 (2003). 14527392 10.1016/j.str.2003.09.005

[b53] Bustamante N., Rico-Lastres P., Garcia E., Garcia P. & Menendez M. Thermal stability of Cpl-7 endolysin from the *Streptococcus pneumoniae* bacteriophage Cp-7; cell wall-targeting of its CW_7 motifs. PLoS One 7, e46654, 10.1371/journal.pone.0046654 (2012). 23056389 PMC3466307

[b54] Bustamante N. *et al.* Cpl-7, a lysozyme encoded by a pneumococcal bacteriophage with a novel cell wall-binding motif. J Biol Chem 285, 33184–33196, 10.1074/jbc.M110.154559 (2010). 20720016 PMC2963342

[b55] Cenens W. *et al.* Expression of a novel P22 ORFan gene reveals the phage carrier state in *Salmonella typhimurium*. PLoS Genet 9, e1003269, 10.1371/journal.pgen.1003269 (2013). 23483857 PMC3573128

[b56] Rashel M. *et al.* Efficient elimination of multidrug-resistant *Staphylococcus aureus* by cloned lysin derived from bacteriophage phi MR11. J Infect Dis 196, 1237–1247, 10.1086/521305 (2007). 17955443

