# Supplementary material for: A chimeolysin with extended-spectrum streptococcal host range found by an induced lysis-based rapid screening method
Source: Sci Rep. 2015 Nov 26;5:17257. doi: 10.1038/srep17257 (PMC4660466; doi:10.1038/srep17257)
Supplement: Supplementary Information [file srep17257-s3.doc]

**Supporting Information**

A chimeolysin with extended-spectrum streptococcal host range found by an induced lysis-based rapid screening method

Hang Yang^1^, Sara B. Linden^2^, Jing Wang^1^, Junping Yu^1^, Daniel C. Nelson^2,3^,*, & Hongping Wei^1^,*

*^1^Key Laboratory of Special Pathogens and Biosafety, Center for Emerging Infectious Diseases, Wuhan Institute of Virology, Chinese Academy of Sciences, Wuhan 430071, China;*

*^2^Institute for Bioscience and Biotechnology Research, University of Maryland, Rockville, MD 20850, USA;*

*^3^Department of Veterinary Medicine, University of Maryland, College Park, MD 20742, USA*

The **Supporting Information** contains the following contents:

**Figure S1 The design and overall steps of the screening method.**

**Figure S2 Effect of IPTG on the growth of host E. coli cells.**

**Figure S3 Images of clearing zones on the chimeolysin screening plates.**

**Figure S4 The characteristics of ClyR.**

**Figure S5 Dose-dependent lytic activity of ClyR and PlyGBS-180.**

**Movie S1: Lysis of *E. coli* (fluorescent channel) during interaction with lysin.**

**Movie S2: Lysis of *E. coli* (bright channel) during interaction with lysin.**

**Table S1 Comparison of the host range of ClyR with several streptococcal lysins.**

**Table S2 Bacterial strains used in this study.**

**Table S3 CD and CBD donors used in this study.**

**Table S4 Primers used in this study.**

**Supplementary References (for Table S1)**

**
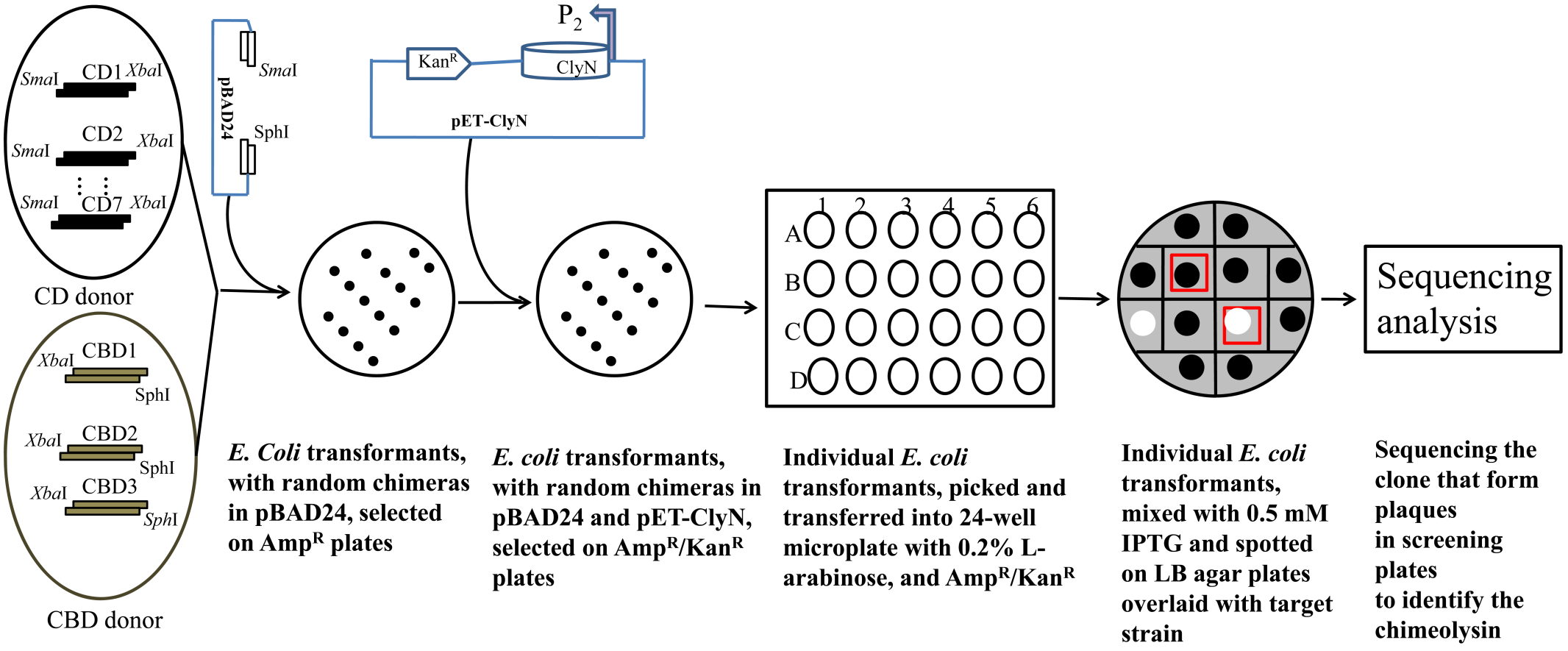
**

**Supplementary Figure S1.** Design and overall steps of the screening method.


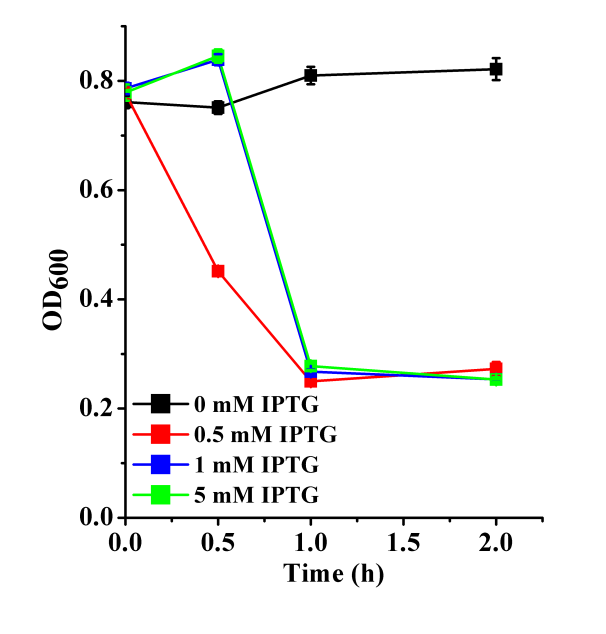


**Supplementary Figure S2. Effect of IPTG on the growth of host *E. coli* cells.** BL21(DE3)/pET-*clyN* cells were cultured in LB to an OD_600_ of 0.8, then induced with different concentrations of IPTG (0, 0.5, 1, and 5 mM) and the changes in OD_600_ were monitored by a microplate reader at different times.


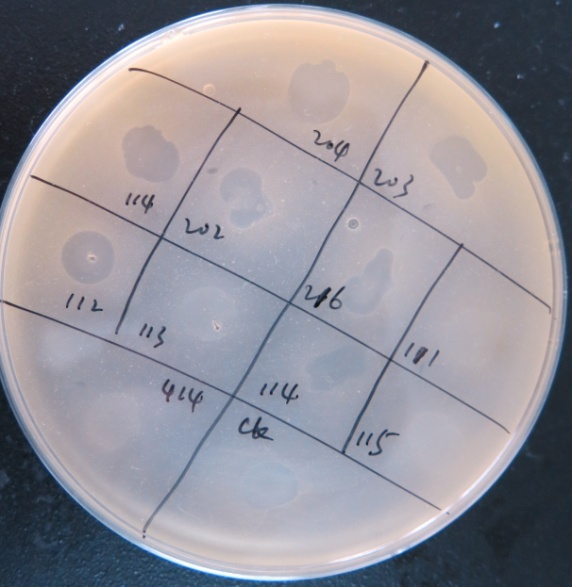


**Supplementary Figure S3. Images of the clearing zones on the chimeolysin screening plates**. After culturing with 0.2% L-arabinose, clones were screened for their capacity for generating clearing plaques on soft agar plates overlaid with *S. dysgalactiae* ATCC 35666.


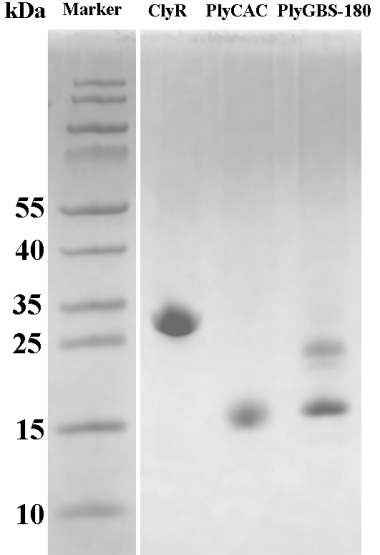

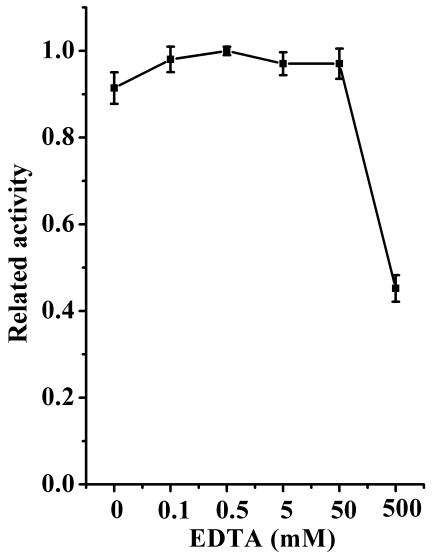

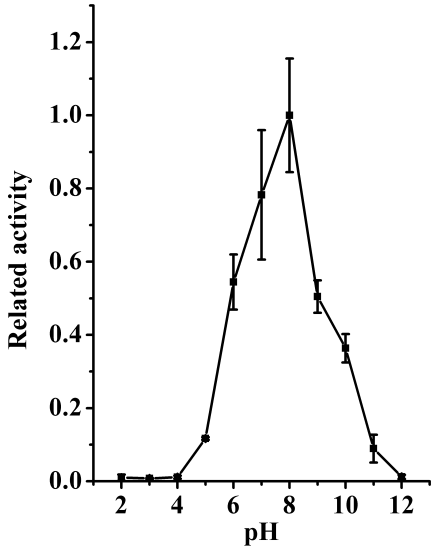


A

B

C


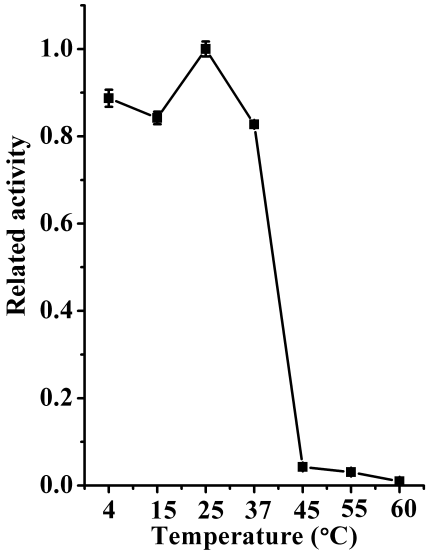

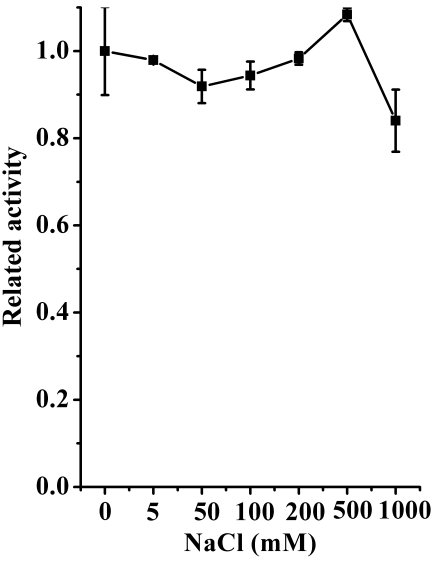

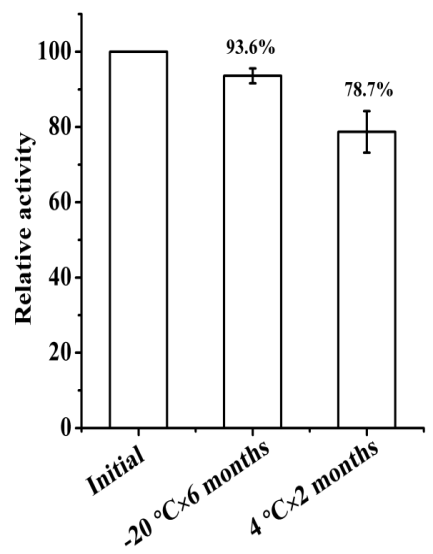


D

E

F

**Supplementary Figure S4. The characteristics of ClyR.** (A) Analysis of purified proteins on 12% SDS-PAGE gel. The influence of EDTA (B), pH (C), temperature (D) and NaCl (E) on the enzymatic activity of ClyR were identified. The lytic activities of ClyR in these conditions were compared and normalized as shown in the graph. (F) Stability of ClyR. The relative activity of ClyR after storing at 4 ºC for 2 months and -20 ºC for 6 months were tested by a microplate reader using *S. dysgalactiae* ATCC 35666 as the indicator strain.


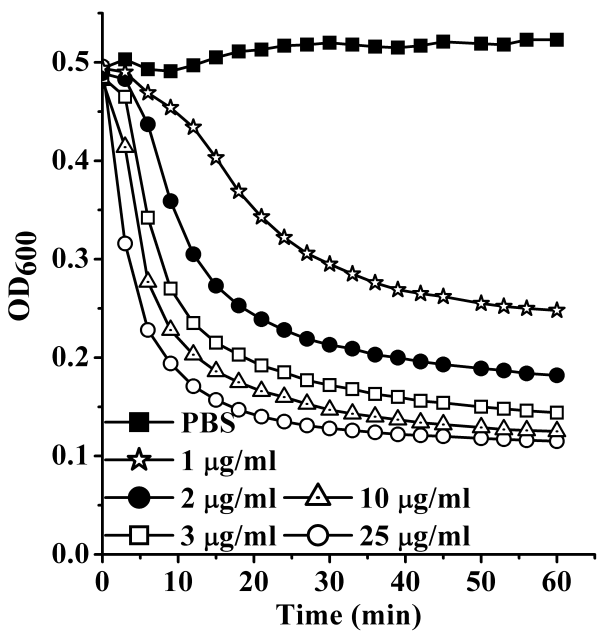

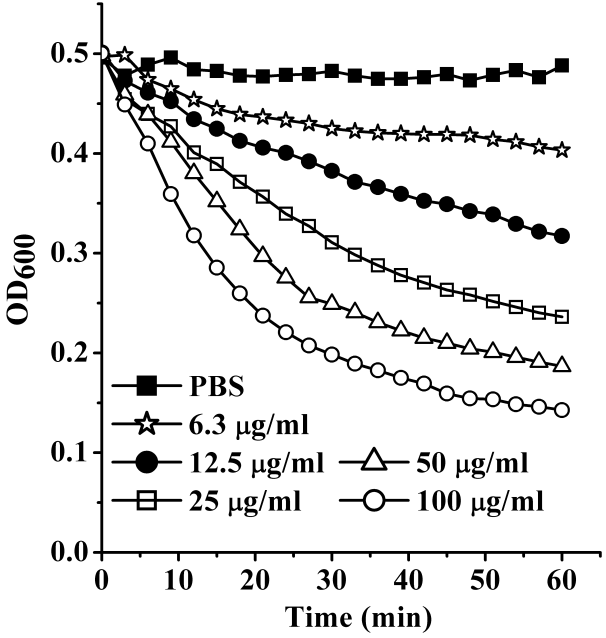


A

B

**Supplementary Figure S5. Dose-dependent lytic activity of ClyR and PlyGBS-180.** *S. dysgalactiae* ATCC 35666 cells were washed once with PBS and then treated with various concentrations of ClyR (A) and PlyGBS-180 (B), the changes of OD_600_ were monitored using a microplate reader at 37 ºC.

**
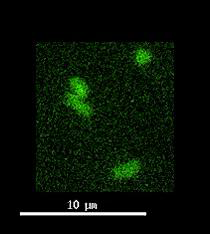
**

**Supplementary Movie S1. Lysis of *E. coli* (fluorescent channel) during interaction with lysin.** BL21(DE3)/pET-*clyN*/pBAD-*egfp* cells were induced with 0.2% L-arabinose, placed onto agar-bases with 1 mM IPTG, then observed immediately by a DeltaVision OMX V4 imaging system. The images in fluorescent channel were noted and combined.

**
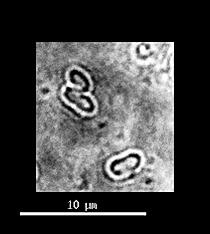
**

**Supplementary Movie S2. Lysis of *E. coli* (bright channel) during interaction with lysin.** BL21(DE3)/pET-*clyN*/pBAD-*egfp* cells were induced with 0.2% L-arabinose, dropped onto agar-bases with 1 mM IPTG, then observed immediately by a DeltaVision OMX V4 imaging system. The images in bright channel were noted and combined.

**Supplementary Table S1. Comparison of the host range of ClyR with several streptococcal** lysins.

| Strains | Host range of several streptococcal lysins^a^ | | | | | | | | | | |
| --- | --- | --- | --- | --- | --- | --- | --- | --- | --- | --- | --- |
|  | ClyR | PlySs2 | PlyC | B30/PlyGBS | PAL | Cpl-1 | Cpl-7 | Ply 700 | Lambda  Sa2 | PlyPy | LySMP |
| *Streptococcus pyogenes* (GAS) | **+** | **+** | **+** | **+** | **-** | **-** | **+** | **+** | **+** | **+** | ND |
| *Streptococcus agalactiae* (GBS) | **+** | **+** | **-** | **+** | ND | **-** | **-** | **+/-** | **+/-** | **+** | ND |
| *Streptococcus dysgalactaie* (GCS) | **+** | **+** | **+** | **+** | ND | **-** | **+/-** | **+** | **+** | **+** | ND |
| *Streptococcus equi* (GCS) | **+** | **+** | **+** | ND | ND | ND | ND | ND | **+** | **+** | **+/-** |
| *Streptococcus uberis* | **+** | ND | **+** | **+** | ND | ND | ND | **+** | **+/-** | **+** | ND |
| *Streptococcus mutans* | **+** | **+/-** | **-** | **+/-** | **-** | **-** | **-** | ND | **+/-** | **+/-** | ND |
| *Streptococcus pneumoniae* | **+** | **+/-** | **-** | **-** | **+** | **+** | **+** | ND | ND | **-** | ND |
| *Streptococcus salivarius* | **+** | ND | **-** | **+** | **-** | **-** | ND | ND | ND | ND | ND |
| *Streptococcus suis* | **+** | **+** | **-** | ND | ND | ND | ND | ND | ND | **+/-** | **+/-** |
| *Streptococcus crista* | **+** | ND | **-** | ND | **-** | **-** | ND | ND | ND | ND | ND |
| *Streptococcus gordonii* | **+** | **+** | **-** | **+/-** | **-** | **-** | ND | ND | ND | **+** | ND |
| *Streptococcus intermidius* | **+** | ND | **-** | ND | **-** | **-** | ND | ND | ND | ND | ND |
| *Streptococcus mitis* | **-** | ND | **-** | ND | **-** | **+/-** | **+** | ND | ND | ND | ND |
| *Streptococcus oralis* | **+/-** | **+/-** | **-** | **-** | **+/-** | **+** | ND | ND | ND | **-** | ND |
| *Streptococcus parasanguinis* | **+** | ND | **-** | ND | **-** | ND | ND | ND | ND | ND | ND |
| *Streptococcus sobrinus* | **+/-** | **+/-** | **-** | **-** | ND | ND | ND | ND | ND | **-** | ND |
| *Streptococcus rattus* | **+/-** | **+/-** | **-** | ND | ND | ND | ND | ND | ND | **-** | ND |
| *Staphylococcus aureus* | **+/-** | **+** | **-** | **-** | ND | **-** | **-** | **-** | **-** | **+/-** | **+/-** |
| *Enterococcus faecalis* | **+** | **+/-** | **-** | **-** | ND | **-** | **+** | ND | ND | **-** | ND |
| *Bacillus cereus* | **-** | **-** | **-** | **-** | ND | ND | ND | ND | ND | **-** | ND |
| Ref. | This work | [1](#_ENREF_1) | [2](#_ENREF_2) | [3-5](#_ENREF_3) | [6](#_ENREF_6) |  | [7](#_ENREF_7) | [9](#_ENREF_9) | [10](#_ENREF_10) | [11](#_ENREF_11) | [12](#_ENREF_12) |

^a^Activity: positive activity (**+**), weak activity (**+/-**), no activity (**-**), or no data collected (ND).

**Supplementary Table S2.** Bacterial strains used in this study.

| Bacteria | Strain | Characteristic | Source^a^ |
| --- | --- | --- | --- |
| Group B streptococci |  |  |  |
| *S. agalactiae* | O90R | no type | 1 |
| *S. agalactiae* | A349 | type IA | 1 |
| *S. agalactiae* | D803 | type IA | 1 |
| *S. agalactiae* | A934 | type IB | 1 |
| *S. agalactiae* | A347 | type II | 1 |
| *S. agalactiae* | A909 | type III | 1 |
| *S. agalactiae* | S12 | no type | 2 |
| Group C streptococci |  |  |  |
| *S. dysgalactiae equisimilis* | 26RP66 |  | 1 |
| *S. dysgalactiae equisimilis* | ATCC 21597 |  | 1 |
| *S. dysgalactiae equisimilis* | ATCC 35666 |  | 3 |
| *S. equi equi* | ATCC 9528 |  | 1 |
| *S. equi* | SE |  | 1 |
| *S. equi* | DB |  | 1 |
| *S. equi* | SL |  | 1 |
| *S. equi* | SF |  | 1 |
| *S. equi* | CF32 |  | 1 |
| *S. equi* | E21 |  | 1 |
| *S. equi* | 90178 |  | 1 |
| *S. equi* | 7-3807-2 |  | 1 |
| *S. equi* | 7-3807-3 |  | 1 |
| *S. equi* | 7-3807-4 |  | 1 |
| *S. equi zooepidemicus* | ATCC 700400 |  | 1 |
| Oral streptococci |  |  |  |
| *S. mutans* | ATCC 25175 | serotype c | 1 |
| *S. mutans* | 10449 | serotype c | 1 |
| *S. mutans* | Kir | serotype d/g | 1 |
| *S. mutans* | B14 | serotype e | 1 |
| *S. mutans* | OMZ 175 | serotype f | 1 |
| *S. mutans* | 2959 | serotype unknown | 1 |
| *S. mutans* | Heicklin | serotype unknown | 1 |
| *S. salivarius* | ATCC 27945 |  | 1 |
| *S. gordonii* | DL1 |  | 1 |
| *S. intermidius* | PK2821 |  | 1 |
| *S. mitis* | J22 |  | 1 |
| *S. oralis* | H1 |  | 1 |
| *S. oralis* | PK34 |  | 1 |
| *S. parasanguinis* | PK2564 |  | 1 |
| *S. crista* | PK1408 |  | 1 |
| *S. sobrinus* | SL1 |  | 1 |
| Mastitic streptococci |  |  |  |
| *S. uberis* | 4 |  | 1 |
| *S. uberis* | 24 |  | 1 |
| *S. uberis* | 27 |  | 1 |
| *S. uberis* | 39 |  | 1 |
| *S. uberis* | 42 |  | 1 |
| *S. uberis* | 72 |  | 1 |
| *S. uberis* | ATCC 27958 |  | 1 |
| Other streptococci |  |  |  |
| *S. pneumoniae* | TIGR4 |  | 1 |
| *S. suis* | 730082 |  | 1 |
| *S. pyogenes* | D471 |  | 1 |
| *S. rattus* | FA-1 |  | 1 |
| Non-streptococcal bacteria |  |  |  |
| *Staphylococcus aureus* | NRS382 |  | 1 |
| *S. aureus* | NRS384 |  | 1 |
| *S. aureus* | NRS385 |  | 1 |
| *S. aureus* | NRS14 | VISA | 1 |
| *S. aureus* | NRS71 |  | 1 |
| *S. aureus* | AM025 | MRSA | 2 |
| *S. aureus* | N315 | MRSA | 2 |
| *S. aureus* | CCTCC AB91118^b^ |  | 2 |
| *S. albus* | ATCC 8799 |  | 3 |
| *Enterococcus faecalis* | EF24 |  | 1 |
| *Bacillus cereus* | 4342 |  | 1 |

^a^Source: Laboratory collection, Institute for Bioscience and Biotechnology Research, University of Maryland, USA. 2. Laboratory collection, Key Laboratory of Special Pathogens and Biosafety, Wuhan Institute of Virology, China. 3. Purchased from Guangdong Culture Collection Center, China.

^b^CCTCC AB91118 is shorted as AB918.

**Supplementary Table S3.** CD and CBD donors used in this study.

| Fragments | Characteristics |
| --- | --- |
| CD donor |  |
| Pc | N-terminal 157 aa of lysin Ply187 (GenBank No.: Y07740.1) |
| LysH5C | N-terminal 300 aa of lysin LysH5 (GenBank: EU573240.1) |
| PlyCAC | N-terminal 153 aa of lysin PlyCA (NCBI No.: NC_004814.1) |
| PlyGBS-180 | N-terminal 180 aa of lysin PlyGBS (GenBank No.: AY428505.1) |
| Ply118C | N-terminal 200 aa of lysin Ply118 (GenBank No.: X85008.1) |
| Ply511C | N-terminal 200 aa of lysin Ply511 (GenBank No.: X85010.1) |
| PlySc | N-terminal 150 aa of lysin PlySs2 (GenBank No.: AGF87539.1) |
| CBD donor |  |
| PlySb | C-terminal 100 aa of lysin PlySs2 (GenBank No.: AGF87539.1) |
| PlyV12b | C-terminal 170 aa of lysin PlyV12 (GenBank No.: AY581208.1) |
| LysAB2b | C-terminal 87 aa of lysin LysAB2 (GenBank No.: HM755898.1) |

**Supplementary Table S4. Primers used in this study**.

| Primers | Sequence (5’-3’) |
| --- | --- |
| Pc-F | TATACCCGGGATGGCACTGCCTAAAAC |
| Pc-R | TTAATCTAGATGGTGGTGTAGGTTTCGGTTC |
| LysH5C-F | TTAACCCGGGATGGAAGTCGCAACGATG-3 |
| LysH5C-R | ATATTCTAGAAGCACATTCTTGGAACGTTG |
| PlyCAC-F | TATACCCGGGATGGCAGCAAATCTGG |
| PlyCAC-R | TATATCTAGATTTGAAGGTAATCAGGCCCGTC |
| PlyGBS180C-F | TTAACCCGGGATGGCTACCTACCAGG |
| PlyGBS180C-R | TATATCTAGAGATCGTTTTGGTCGTGC |
| Ply118C-F | TATACCCGGGATGACCTCATACTGCTATTC |
| Ply118C-R | TATATCTAGATTTACCGGCTTTCAGTTTCG |
| Ply511C-F | AATTCCCGGGATGGTCAAATACACGGTG |
| Ply511C-R | TATATCTAGACAGATTCGTAGACGGGGTGC |
| PlySC-F | TTAACCCGGGATGACAACAGTAAATGAAG |
| PlySC-R | TTAATCTAGATGTGATGTAATGCACGACCTG |
| PlySB-F | TATATCTAGATCTCGTTCCTATCGCGAG |
| PlySB-R | TATAGCATGCTTATTTAAATGTACCCCAAG |
| PlyV12B-F | ATATTCTAGATTAAACGGTGGAAGCAC |
| PlyV12B-R | TATAGCATGCTTACTTAAATGTACCCCATG |
| LysAB2B-F | TATATCTAGAAATCCGGAAAAAGCTCTG |
| LysAB2B-R | TATAGCATGCTTACAGAGAGCGCAGAG |
| EGFP-F | TATACCCGGGATGGTGAGCAAGGGCGAG |
| EGFP-R | TATAGCATGCTTACTTGTACAGCTCGTCCATG |
| ClyR-F | TATACCATGGGCATGGCAGCAAATCTGG |
| ClyR-R | ATATCTCGAGTTTGAAGGTACCCCATGCGTTG |
| PlyCAC-R | ATATCTCGAGTTTGAAGGTAATCAGG |
| PlyGBS-180-F | TATACATATGATGGCTACCTACCAGGAATAC |
| PlyGBS-180-R | ATATCTCGAGGATCGTTTTGGTCGTGC |

**Supplementary References (for Supplementary Table S1)**

1 Gilmer, D. B., Schmitz, J. E., Euler, C. W. & Fischetti, V. A. Novel bacteriophage lysin with broad lytic activity protects against mixed infection by *Streptococcus pyogenes* and methicillin-resistant *Staphylococcus aureus*. *Antimicrob Agents Chemother* **57**, 2743-2750, doi:10.1128/AAC.02526-12 (2013).

2 Nelson, D., Loomis, L. & Fischetti, V. A. Prevention and elimination of upper respiratory colonization of mice by group A streptococci by using a bacteriophage lytic enzyme. *Proc Natl Acad Sci U S A* **98**, 4107-4112, doi:10.1073/pnas.061038398 (2001).

3 Donovan, D. M. *et al.* Peptidoglycan hydrolase fusions maintain their parental specificities. *Appl Environ Microbiol* **72**, 2988-2996, doi:10.1128/AEM.72.4.2988-2996.2006 (2006).

4 Cheng, Q., Nelson, D., Zhu, S. & Fischetti, V. A. Removal of group B streptococci colonizing the vagina and oropharynx of mice with a bacteriophage lytic enzyme. *Antimicrob Agents Chemother* **49**, 111-117, doi:10.1128/AAC.49.1.111-117.2005 (2005).

5 Pritchard, D. G., Dong, S., Baker, J. R. & Engler, J. A. The bifunctional peptidoglycan lysin of *Streptococcus agalactiae* bacteriophage B30. *Microbiology* **150**, 2079-2087, doi:10.1099/mic.0.27063-0 (2004).

6 Loeffler, J. M., Nelson, D. & Fischetti, V. A. Rapid killing of *Streptococcus pneumoniae* with a bacteriophage cell wall hydrolase. *Science* **294**, 2170-2172, doi:10.1126/science.1066869 (2001).

7 Diez-Martinez, R. *et al.* Improving the lethal effect of cpl-7, a pneumococcal phage lysozyme with broad bactericidal activity, by inverting the net charge of its cell wall-binding module. *Antimicrob Agents Chemother* **57**, 5355-5365, doi:10.1128/AAC.01372-13 (2013).

8 Loeffler, J. M., Djurkovic, S. & Fischetti, V. A. Phage lytic enzyme Cpl-1 as a novel antimicrobial for pneumococcal bacteremia. *Infect Immun* **71**, 6199-6204 (2003).

9 Celia, L. K., Nelson, D. & Kerr, D. E. Characterization of a bacteriophage lysin (Ply700) from *Streptococcus uberis*. *Vet Microbiol* **130**, 107-117, doi:10.1016/j.vetmic.2007.12.004 (2008).

10 Donovan, D. M. & Foster-Frey, J. LambdaSa2 prophage endolysin requires Cpl-7-binding domains and amidase-5 domain for antimicrobial lysis of streptococci. *FEMS Microbiol Lett* **287**, 22-33, doi:10.1111/j.1574-6968.2008.01287.x (2008).

11 Lood, R., Raz, A., Molina, H., Euler, C. W. & Fischetti, V. A. A highly active and negatively charged *Streptococcus pyogenes* lysin with a rare D-alanyl-L-alanine endopeptidase activity protects mice against streptococcal bacteremia. *Antimicrob Agents Chemother* **58**, 3073-3084, doi:10.1128/AAC.00115-14 (2014).

12 Wang, Y., Sun, J. H. & Lu, C. P. Purified recombinant phage lysin LySMP: an extensive spectrum of lytic activity for swine streptococci. *Curr Microbiol* **58**, 609-615, doi:10.1007/s00284-009-9379-x (2009).
